# How do goats “read” 2D-images of familiar and unfamiliar conspecifics?

**DOI:** 10.3389/fpsyg.2023.1089566

**Published:** 2023-05-19

**Authors:** Jan Langbein, Mauricio Moreno-Zambrano, Katrin Siebert

**Affiliations:** ^1^Research Institute for Farm Animal Biology, Institute of Behavioural Physiology, Dummerstorf, Germany; ^2^Research Institute for Farm Animal Biology, Institute of Genetics and Biometry, Dummerstorf, Germany

**Keywords:** domestic ungulates, visual discrimination, reversal learning, face recognition, individual recognition

## Abstract

To study individual recognition in animals, discrimination tasks are often conducted by presenting 2D images of real conspecifics. However, animals may discriminate the images merely as visual stimulus combinations without establishing referential relationships to the individuals depicted. In the current study, we investigated whether goats are able to discriminate photos of familiar and unfamiliar conspecifics, whether they not only process the photos as visual stimuli, but also understand them as virtual copies of real conspecifics and whether they grasp the concept of familiarity. Using a computer-controlled learning device, in three tests, goats of two experimental groups (A and B) had to discriminate portrait (Te1), profile (Te2) or headless body photos (Te3) of conspecifics. Tests were presented as 4-choice tasks, with one photo from Group A (rewarded) plus three photos from Group B (distractors). That is, the rewarded photo was familiar to Group A, but unfamiliar to Group B. Finally, in a reversal test (Te4) we reversed this principle. The goats learned the discriminations in Te1 to Te3 within two (Te1 and Te2) and three training days (Te3), respectively, and they needed between 91 [CL (66, 126)] and 174 [CL (126, 241)] trials to reach the learning criterion, with no statistically significant differences between the groups. In Te4, in contrast, the animals took 403 [Group A; CL (291, 557)] and 385 [Group B; CL (286, 519)] trials, respectively, to learn the task. The lack of spontaneous preferences for the photo of the familiar conspecific in the pretests of Te1 to Te3 in Group A, as well as the lack of differences in the number of trials to learn the discriminations between both groups, do not at first glance suggest that the goats established a correspondence between real conspecifics and their 2D representations. However, the higher number of trials in Te4 suggests that both groups formed the learning rule of choosing either the known (Group A) or the unknown goat (Group B) over the course of Te1 to Te3 and then failed after the rule was reversed, providing evidence that goats can associate 2D photos of conspecifics with real animals.

## 1. Introduction

Social recognition is based on the process of dividing conspecifics into different categories, such as homo-vs. heterospecific, young vs. adult, female vs. male, kin vs. nonkin, dominant vs. subordinate, and familiar vs. unfamiliar (Gheusi et al., 1994). These different levels of distinctiveness describe an ascending continuum from simpler to increasingly complex social recognition. Along this continuum, the ability to recognize conspecifics has been categorized into class-level recognition or individual recognition, and the latter might be seen as a special case of the former (Tibbetts and Dale, 2007; Yorzinski, 2017). Two different categories of class-level recognition have been described: (i) “receivers learn the signaller’s individually distinctive characteristics and associate these characteristics with inferred class-specific information about the signaller,” or (ii) “receivers match the signaller’s phenotype to an internal template associated with different classes” (Tibbetts and Dale, 2007). Grouping conspecifics into classes (categories) reduces the enormous amount of information in a complex social environment and enables the organism to react efficiently to a specific individual (Zayan and Vauclair, 1998; Ghirlanda and Enquist, 2003; Lombardi, 2008).

For individual recognition (IR) subjects learn the individually distinctive phenotype of conspecifics (signature), store their mental representations as a single natural category (prototype) and assign specific properties to this individual (Zayan, 1994; Yorzinski, 2017). IR is the cornerstone for complex social behaviour in many different taxa, including insects (Tibbetts, 2002), amphibians (Martin et al., 2020), reptiles (Carazo et al., 2008), birds (Brecht et al., 2020), and mammals (Kendrick, 2006) as it allows individuals to quickly adapt their behaviour to the conspecifics they encounter at any given time. While IR is involved in diverse social contexts, the main focus of IR research is on familiarity, dominance interactions, pair bonding and parental care (Tibbetts and Dale, 2007; Cely and Tibbetts, 2017). For IR, species use different sensory modalities, such as chemical, visual, and auditory cues, or a mixture of the different modalities to build a mental representation of a conspecific (Lampe and Andre, 2012). Some of these modalities can be used for long-distance recognition (Briseño-Jaramillo et al., 2015), while others are effective for close-range interactions (Tibbetts, 2002). In many vertebrate taxa that rely mainly on vision for social recognition, the key features are often size, posture, ornamentation or body appendages such as feathers, horns or antlers (Walther, 1984), while preferences for facial cues have been noted for IR (Leopold and Rhodes, 2010).

To study IR and the role of facial stimuli in this process, 2D images of real conspecifics are often used, either printed in different sizes or, more recently, presented on a computer screen. Presenting images is advantageous because other modalities, such as chemical or acoustic stimuli, can be excluded. Images of conspecifics as 2D presentations can be discriminated based on different cognitive processes. Animals might understand the images as visual stimuli without reference to the real subjects that are depicted, take the images for real subjects themselves, or finally interpret the images as a depiction of real subjects. According to Fagot et al. (2000) these three distinct processes are referred to as (i) independence, (ii) confusion, or (iii) equivalence. Therefore, the simple discrimination of visual stimuli based on (i) is the prerequisite for the subsequent true IR (iii) (Brajon et al., 2015). Fagot et al. (2010) defined this as either the “*low road”* or *“high road”* of processing “repeatedly experienced pictorial stimuli depicting animate or inanimate objects encountered in life.”

Since Herrnstein’s seminal work on picture discrimination and categorization in pigeons (Herrnstein, 1964), the ability to discriminate pictures has been demonstrated in many different taxa, from insects to nonhuman primates (Bovet and Vauclair, 2000). However, most of these studies did not verify whether the animals actually interpreted the depicted objects as images of the real 3D objects or merely learned to distinguish them as arbitrary visual stimuli. Dasser (1987) investigated this question in long-tailed macaques (*Macaca fascicularis*). Three subjects were first asked to discriminate group members on slides. Next, they matched different views of the same subject. Finally, one subject, in a matching to sample (MTS) test, matched new slides of known and unknown conspecifics to the corresponding categories in 1st trial. Although only three animals were trained and tested, the experiments showed that the monkeys could match pictures with real conspecifics and assign them to classes such as familiar and unfamiliar. Dittrich (1994), using a 4-choice discrimination approach, trained long-tailed macaques (*Macaca fascicularis*) to discriminate line drawings of different monkey bodies. The animals learned the discrimination within 70 to 490 trials. They also matched different pictures of the same individual even if they differed in size and orientation. The authors revealed generalization to different views of facial stimuli (frontal or lateral). Another study (Pokorny and de Waal, 2009) used a similar approach in capuchin monkeys to investigate whether they can discriminate between familiar and unfamiliar conspecifics on slides and interpret them as pictures of real conspecifics. The animals first learned to distinguish one in-group member from three out-group members or vice versa by means of an oddity task. In transfer trials, the animals applied the learned concept both to new pictures of the same subjects and to pictures of juveniles never presented before. These studies demonstrate that different nonhuman primate species can understand the referential relationship between drawings or photos of conspecifics and real individuals by demonstrating picture equivalence (Pokorny and de Waal, 2009).

Sophisticated picture processing skills have also been demonstrated in domesticated animals. In addition to studies in chickens (Rosa-Salva et al., 2010), horses (Proops and McComb, 2010; Baba et al., 2019) or pigs (Brajon et al., 2015; Wondrak et al., 2018) in this field, and with a particular focus on the IR of conspecifics, the studies by Kendrick in sheep and Coulon in cattle are noteworthy. Sheep discriminate pictures of conspecifics of the same sex based on facial cues (Kendrick et al., 1995) and they discriminated faces of sheep faster than geometric patterns as well as faces of sheep of a known breed faster than faces of sheep of an unknown breed (Kendrick et al., 1996). Kendrick interpreted these results as a prerequisite for the brain’s ability to store a mental prototype of another individual’s characteristics (Zayan, 1994), thus enabling IR. However, as far as we know, Kendrick never performed transfer experiments such as those described in the monkey papers mentioned above to demonstrate that the sheep actually formed the categories familiar and unfamiliar based on IR. Coulon et al. (2007) showed in a first experiment that heifers (*Bos taurus*) can learn to visually discriminate images of different cattle breeds from images of other domestic animal species. Some heifers reached the learning criterion in training after only 50 trials and in a generalization follow-up test after 80 trials, although the performance of the heifers varied greatly depending on the subjects. In a second study, Coulon et al. (2009) showed that heifers are capable of classifying different 2D views of the same conspecific into a single category. This was easier for the animals if they were socially familiar with the individuals depicted. This was interpreted as evidence of the ability of IR in cattle. Finally, a third study (Coulon et al., 2010) examined the ability of cattle to discriminate between facial views of known and unknown conspecifics presented as 2D pictures. The spontaneous response to the pictures was analysed in a pretest (PT), before the actual training began. In the PTs, heifers were more interested in the picture of a familiar conspecific than in the picture of an unfamiliar conspecific. The majority of trained heifers were able to discriminate between pictures of familiar and unfamiliar conspecifics and generalize to new pictures falling into these categories on the first trial. The authors concluded that with regard to IR based on visual cues, cognitive capacities in cattle match those shown in other species (Dasser, 1987; Kendrick et al., 1996; Bird and Emery, 2008; de Waal and Pokorny, 2008; Pokorny and de Waal, 2009; Sheehan and Tibbetts, 2011).

In previous studies, we examined the visual learning abilities of dwarf goats using a 4-choice visual discrimination task presented by a fully automated learning device with a 17 inch monitor. The learning device was integrated into the goats’ home pen, and the goats had 24/7 access to it, so they decided when and for how long they wanted to use it. With this experimental design, we avoided any human influence on learning and an influence of lack of current motivation on learning performance. We have demonstrated that goats can discriminate between simple and complex visual stimuli (Langbein et al., 2006), that they improve their learning performance in successive tasks of the same type (Langbein et al., 2007), and that they can retain successively learned sets of visual stimuli over several weeks and later recall them concurrently (Langbein et al., 2008). We demonstrated that goats are able to form categories based on similarities in the visual appearance of artificial symbols and to generalize across new symbols (Meyer et al., 2012). Goats have shown that they have an intrinsic interest in such learning tasks that goes beyond the reward (Langbein et al., 2009), and we found that the combination of structural and cognitive enrichment in particular can improve the behavioural competence of dwarf goats in challenging situations (Oesterwind et al., 2016).

In the current study, we investigated whether goats are able to discriminate between familiar conspecifics from their own group and unfamiliar goats based on photos, as has previously been shown in several primate species (Dasser, 1987; Pokorny and de Waal, 2009) and farm animals such as cattle and sheep (Kendrick et al., 1996; Coulon et al., 2010). The discrimination paradigm is comparable to that used by Pokorny and de Waal (2009) in monkeys. In three different tests, goats of experimental groups A and B had to distinguish a photo of a goat from group A (S^+^) from three photos of goats from group B (S^−^, distractors). That is, the rewarded photo was familiar to Group A but unfamiliar to Group B. In a fourth test we reversed this principle. Now the rewarded photo was of a goat from Group B, and the other three photos were of goats from Group A. We applied a fully automated learning device to present the photos as 4-choice visual discrimination tests (see above). Computer-controlled testing excluded the influence of human experimenters and any cues other than visual ones (Wondrak et al., 2018). In all four tests the same photos were shown to the animals of both groups and in each test photos of new goats were used. To test for spontaneous preferences for a particular photo, each test was preceded by a 1 day pretest in which any choice was rewarded, followed by 6 days to learn the tests.

In accordance with previous work on the concept of familiarity in other species (Sugita, 2008; Coulon et al., 2010; Meary et al., 2014), we expected a spontaneous preference for photos of familiar conspecifics in the pretest. During training, we expected Group A to show higher learning performance than Group B because this group had to discriminate the photo of a known goat, whereas for Group B the rewarded photo was an unknown goat. Similar results were found in primates asked to discriminate known from unknown conspecifics based on 2D photos (Talbot et al., 2015, 2016). Finally, we expected that the goats’ learning performance in successive learning tests would improve by leaps and bounds if they not only distinguished the photos as visual stimuli, but also understood them as virtual copies of real subjects and grasped the concept of familiarity.

## 2. Animals materials and methods

### 2.1. Animals and experimental groups

The study was conducted at the Research Institute for Farm Animal Biology (FBN), in Dummerstorf, Germany. We used 28 female Nigerian dwarf goat kids (*Capra aegagrus hircus*) from a line bred at our institute. Until weaning, the animals were kept in two spatially separated groups of up to 25 adult females with their kids of both sexes. In the housing pen, we offered straw as bedding, concentrate twice daily, and hay and water *ad libitum*. After weaning, at a mean age of 55 days, 14 infants from one group were assigned to experimental group A and 14 from the other group to experimental group B. Both groups were kept in indoor pens (24 m^2^) equipped with straw as bedding, a two-floor wooden climbing rack, a round feeder for concentrate (120 g/day/animal), and a hay rack (hay *ad libitum*). Each pen had a separate compartment, not visible from the outside, where the learning device was installed.

### 2.2. Learning device

The learning device consisted of a 17″ flat stainless P-CAP IP65 touchscreen with cover glass (Bressner Technology GmbH, München) and additional protective foil (Folierbar, Rostock). The touchscreen was connected to a desktop computer. For presentation of visual stimuli, up to 12 sensitive fields can be defined on the touchscreen. For identification at the learning device, the goats were equipped with an electronic transponder (TECTUS Transponder Technology GmbH, Moers), which was recognized by a rectangular RFID antenna (TECTUS Technology GmbH, Bergisch Gladbach) in front of the touchscreen. We used control software from HaSoTec (Rostock, Germany) to present the stimuli, register the actions of the goats on the learning device, and provide a reward as a primary reinforcer for a correct choice.

**Figure 1:**
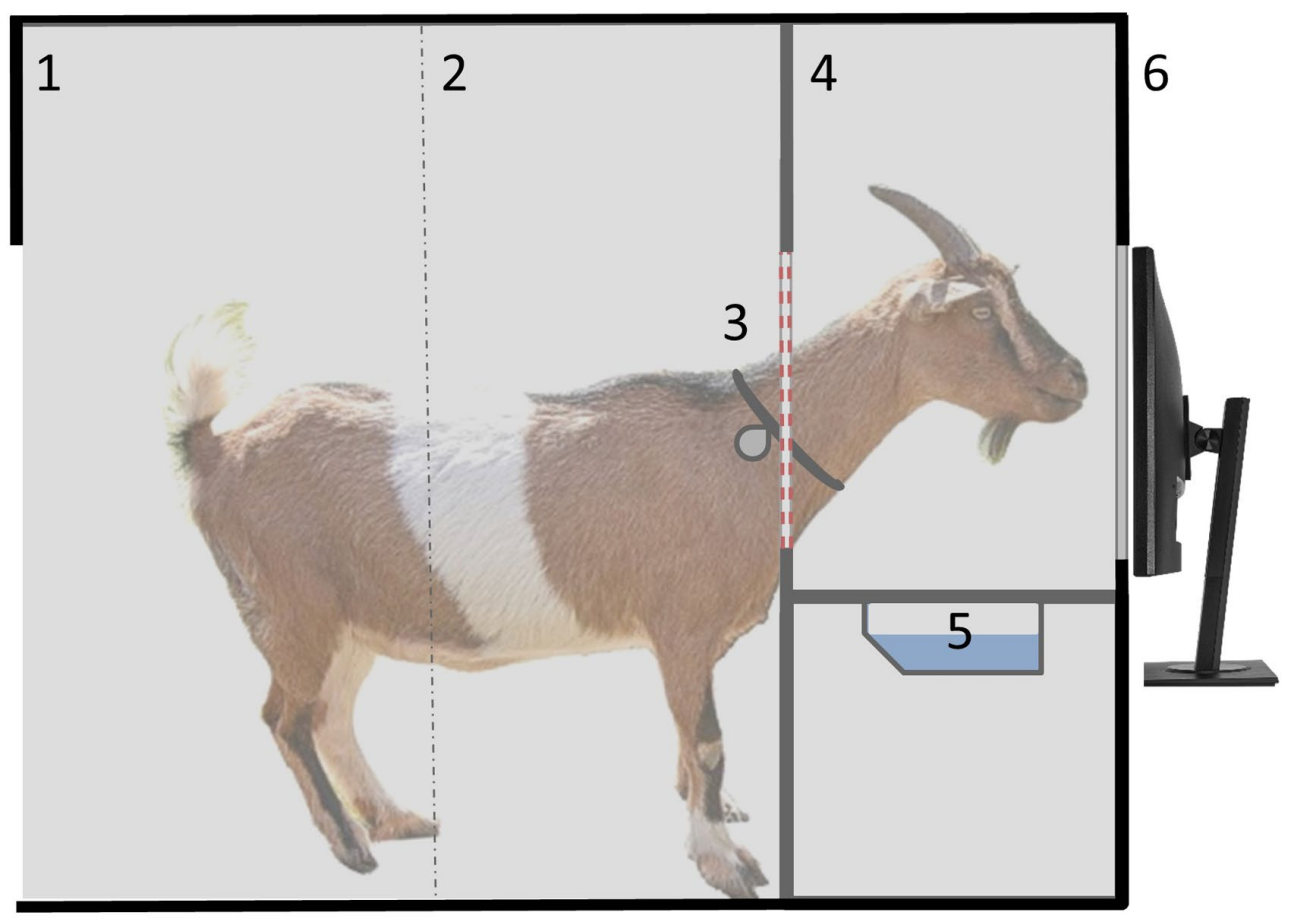
Lateral view of a goat inside the compartment with the learning device: 1 = entrance (only one goat could enter at a time to avoid observational learning by pen mates), 2 = light beam to indicate when a goat entered/left the device, 3 = collar with RFID transponder for individual identification at the device, 4 = yoke to put the head through plus integrated RFID antenna, 5 = water bowl for reward delivery, and 6 = touchscreen for stimuli presentation.

In addition to the reward, two acoustic secondary reinforcers were used, which differed on correct (tone A: 440 Hz; 80 db) and incorrect trials (superposition of tones B, C and D: 980, 1,039, 1,166 Hz; 80 db). The device was housed in a separate compartment that could not be seen from the outside, and was accessible to the goats 24/7 (Figure 1). Due to the limited dimensions of the compartment, the device could only be used by one animal at a time. The goats were free to decide when to visit the learning device and how many actions they wanted to perform during a visit. Drinking water (30 mL) was used as a reward for a correct choice (Langbein et al., 2007).

### 2.3. Shaping

We shaped the goats stepwise to the learning device. We started with a float switch hanging in the water bowl to keep the bowl half-filled while a red dot was displayed on the touchscreen just behind the switch. By touching the red dot with its nose, the goats could add an additional 30 mL of water to the bowl. After 3 days, the float switch was removed and the goats had to touch the red dot to add 30 mL of water to the bowl. After eight more days the red dot was presented 20 cm above the bowl in the middle of the screen and had to be touched to add water to the bowl (for 3 days). Finally, we presented two red dots 20 cm above the bowl, and approximately 23 cm apart. On the first 2 days the left button and on the next 2 days the right button had to be touched to add water into the bowl. For the next 4 days, the rewarded dot changed daily. By the end of the shaping phase, all goats had established an association between the red dot and water delivery, and were able to meet their daily water needs (approximately 1 L).

### 2.4. Training

**Figure 2:**
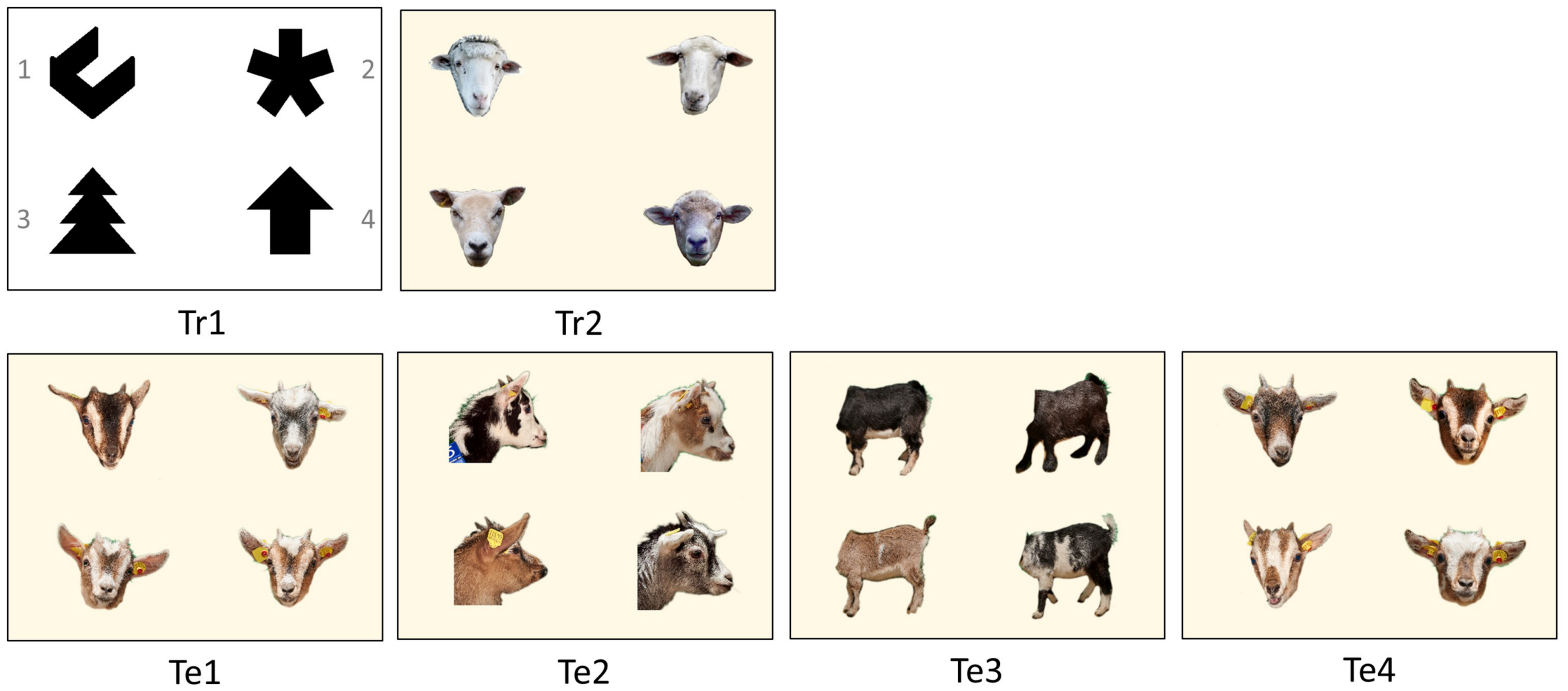
Visual four-choice discrimination problems for training (Tr) and for the tests (Te). The rewarded photo (S^+^) within each test is placed in the upper left corner in this example. After each trial, the photos switched positions on the monitor. The numbering of the photos is given in Tr1. In Te1 to Te3, the rewarded photo was a goat from Group A (S^+^), while the three other photos were goats from Group B (S^−^). In Te4, this principle was reversed. It featured one photo from Group B (S^+^) and three photos from Group A (S^−^).

During training, the goats were presented with two 4-choice discrimination tasks on the touchscreen, one after the other. Each task was trained for 14 days. For the first task (Tr1, Figure 2), we used four black shapes against white background (open square, fir tree, asterisk, and upright arrow). The shapes were presented in sensitive fields of 7 × 7 cm, so that the screen was divided into 4 virtual sectors. To obtain a water reward, the goats had to discriminate the shape that was predetermined to be the S^+^ (open square) from the three distractors by touching it with its nose. Each trial was followed by an intertrial interval (ITI) of 3 s of a black screen before the shapes were shown rearranged in the next trial. The arrangement of the shapes in consecutive trials followed a pseudorandom series. This series consisted of two pseudorandomized subsets of all 24 possible combinations of the shapes. By this series, we ensured that the S^+^, as well as the three distractors (S^−1^, S^−2^, and S^−3^), were equally distributed in the four positions. The controlling software ensured that any side or positional tendencies that the goats might develop were counteracted at all times (Langbein et al., 2006).

For the second task (Tr2, Figure 2), we used four colour portrait photos of different sheep heads. The original background of the images was removed and replaced by the same uniform light yellow background (Power Point, RGB, 240, 235, 215, and 50% transparency). The task was presented in the same way as Tr1. At the end of training, all goats were able to use the device properly and reached the defined learning criterion (please see the *Data analysis and statistics* section for further details). To proceed with only equally well pretrained animals, the two goats with the lowest learning performance in Tr2 were removed from both groups and all further tasks were performed with 12 animals per group.

### 2.5. Discrimination of 2D presentations of familiar versus unfamiliar conspecifics

#### 2.5.1. Stimuli preparation

As stimuli for the following tests, six portrait photos, six profile photos of the head and six headless body photos were taken of each of the 24 goats. The goats were habituated to the shooting environment for 2 weeks to avoid stress during the photo session. The six photos of the different series were slightly different. The original background was removed from the photos and replaced with a uniform neutral background (see above).

#### 2.5.2. Tests

In all four tests, the same photos were shown to the animals in both groups. In each test, photos of new goats were used. The stimuli sets for the first three tests (Te1-3, Figure 2) always consisted of photos of four different goats, one of Group A (S^+^, rewarded) and three of Group B (S^−^, distractors). The sets consisted of portrait photos (Te1), profile photos (Te2) and headless body photos (Te3). The fourth test (Te4) was a reversal test. We used four portrait photos again, but one goat of Group B (S^+^, rewarded) and three goats of Group A (S^−^, distractors). This means that in Te1 to Te3, goats from Group A had to discriminate one familiar from three unfamiliar goats and goats from Group B had to discriminate one unfamiliar goat from three familiar ones. In Te4 this principle was reversed. In all four tests, we used six slightly different photos of each new goat. The size of the individual photos on the screen was 7 × 7 cm.

The arrangement of the individual photos in the successive trials in each test and the presentation of the successive trials were performed similarly to the training. In each test, however, we used six slightly different photos of each goat four times in a series of the 24 possible combinations of the stimuli. Each test was run for 7 days. On the first day, all choices were rewarded, regardless of which photo was chosen. These one-day pretests (PT1 to PT4) served to identify possible spontaneous preferences for a particular photo. On the following 6 days, only touching the predefined correct photo (S^+^) was rewarded with 30 mL of drinking water.

### 2.6. Data analysis and statistical procedures

Because single goats were separated from the rest of the group and undisturbed while acting at the learning device, and the compartment with the device was surrounded by opaque walls to avoid any form of social learning, we treated individuals within the group as independent replicates for statistical purposes (Langbein et al., 2007). In each test, we excluded the goats that were presented with an image of themselves, either as a reward stimulus or as a distractor. That is, in Te 1–3, one animal in Group A and three animals in Group B were excluded from the analysis of the data. In Te 4 this ratio was reversed, three animals from group A and one from group B were excluded.

To check whether there were any preferences for certain photos in the different stimuli sets in the one-day PTs and whether there were differences in the preferences between Groups A and B, we analysed the percentage of choices (*%Choice*) for each of the four photos. We conducted unbalanced two-way ANOVAs for the different PTs, with *Photo* and *Group* and their interaction as explanatory variables. Before conducting ANOVA, we checked the data for homogeneity of variance (Levene-Test), and for normality of the residuals. As the homogeneity of variance assumption was not satisfactorily met in PT1 and PT2 we successfully applied Box-Cox transformation. We visually inspected the normality of the residuals produced by the ANOVA applied to each PT. Least squared means (LSMs) and their confidence levels (CLs) were computed for the explanatory variables.

To get an idea of the learning curves over the six training days, we calculated the daily learning success in Te1–Te4. To analyse the absolute learning performance, we calculated the number of trials that individual goats needed to reach the learning criterion (TtC). The learning criterion was defined as 46% of correct choices in at least two consecutive sequences of 20 trials (*p* < 0.05; binomial test; *N* = 20; *P*0 = 0.25; Hanggi, 1999; Langbein et al., 2007). After conducting the Levene-test on the original *TtC* data, we applied a logarithmic transformation to satisfy the assumption of homogeneity of variances. We used a linear mixed model (LMM) to analyse *TtC* using *Test, Group,* and their interaction as explanatory variables. The model included the animal as a repeated factor in the random statement. We conducted a type III ANOVA for the LMM to produce an ANOVA table for fixed-effect terms. We used Satterthwaite methods for denominator degrees of freedom for F tests. LSMs and CLs were computed for the explanatory variables. We conducted *post hoc* tests of subclasses to find significant differences between *Tests*, *Groups* or their interaction with a Tukey honestly significant difference correction. Mean differences with *p* < 0.05 were considered as statistically significant in all tests.

## 3. Results

### 3.1. Preference test

**Figure 3:**
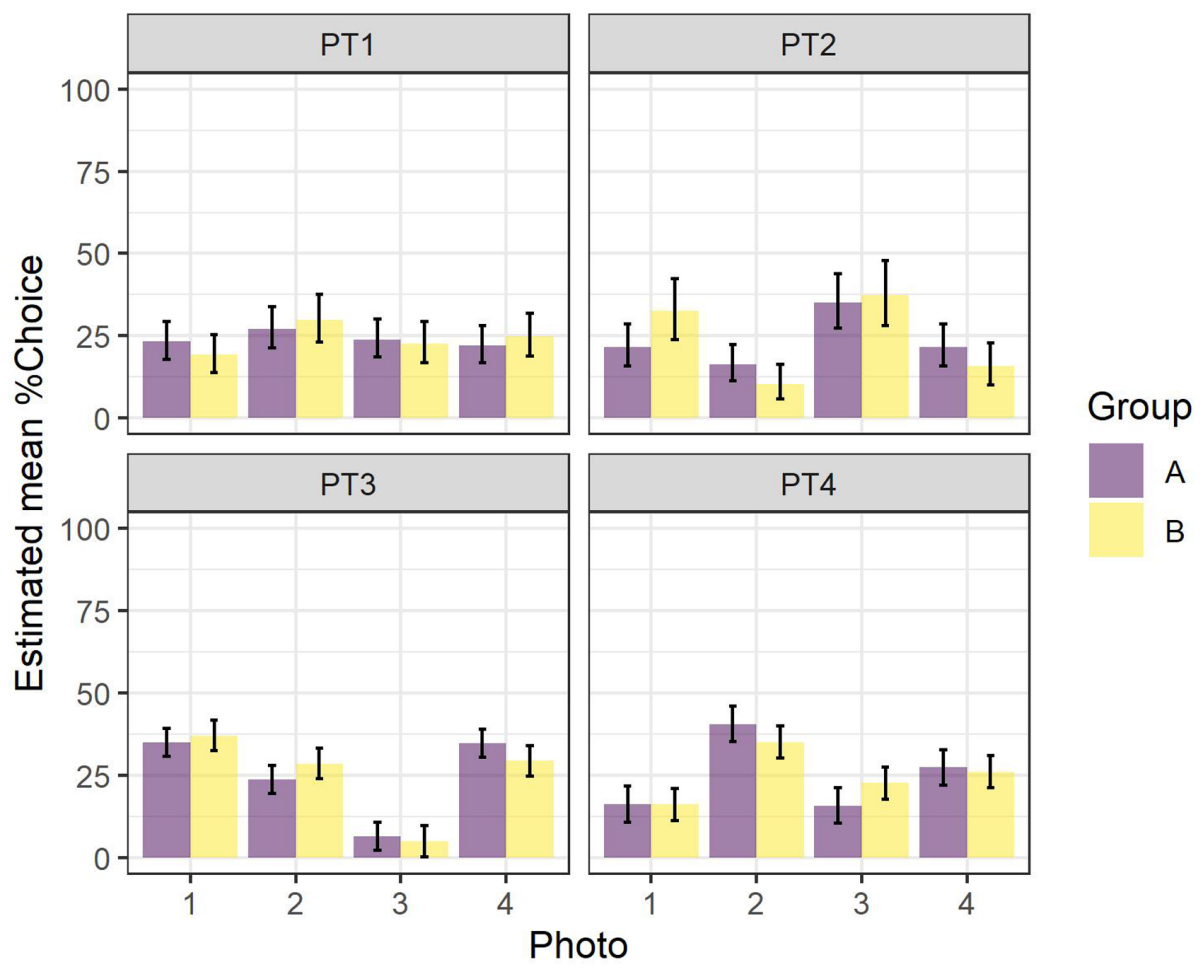
Estimated mean percentage of choice (*%Choice*, LSM ± CL) of the different photos in Group A and Group B for PT1 to PT4.

The results of the two-way ANOVAs for the 1 day pretests (PT1 to PT4) of the impact of *Photo* and *Group* on the *%Choice* of the different photos are given in Table 1. With the exception of PT1, there were statistically significant differences in the preferences for various photos in the different stimuli sets. There were no differences between the two groups on these preferences in any of the pretests. There were also no differences in the interaction between stimuli and group. The estimated mean *%Choice* of the different photos and differences between them are shown in Figure 3. In none of the PTs did we find a preference for the later rewarded photo 1. In PT4, there was a preference for photo 2, at least in Group A, and in PT 3, there was a strong tendency in both groups to avoid choosing photo 3.

**Table 1:** Results of the two-way ANOVA to check for preferences for certain photos in the one-day PTs and for differences in the preferences between Groups A and B.

	Sum Sq	D*f*	*F* value	Pr(>*F*)
*Anova Table PT1*				
(Intercept)	4,849.63	1.00	1,250.03	0.000
Group	0.01	1.00	0.00	0.967
Photo	23.19	3.00	1.99	0.123
Group:Photo	6.93	3.00	0.60	0.620
Residuals	279.33	72.00		
*Anova Table PT2*				
(Intercept)1	4,273.27	1.00	817.31	0.000
Group	0.31	1.00	0.06	0.807
Photo	238.78	3.00	15.22	0.000
Group:Photo	41.32	3.00	2.63	0.057
Residuals	355.54	68.00		
*Anova Table PT3*				
(Intercept)	49,500.00	1.00	990.08	0.000
Group	0.00	1.00	0.00	1.000
Photo	10,789.86	3.00	71.94	0.000
Group:Photo	292.90	3.00	1.95	0.129
Residuals	3,599.72	72.00		
*Anova Table PT4*				
(Intercept)	49,500.00	1.00	749.40	0.000
Group	0.00	1.00	0.00	1.000
Photo	5,530.71	3.00	27.91	0.000
Group:Photo	397.02	3.00	2.00	0.121
Residuals	4,755.78	72.00		

### 3.2. Discrimination tests

**Figure 4:**
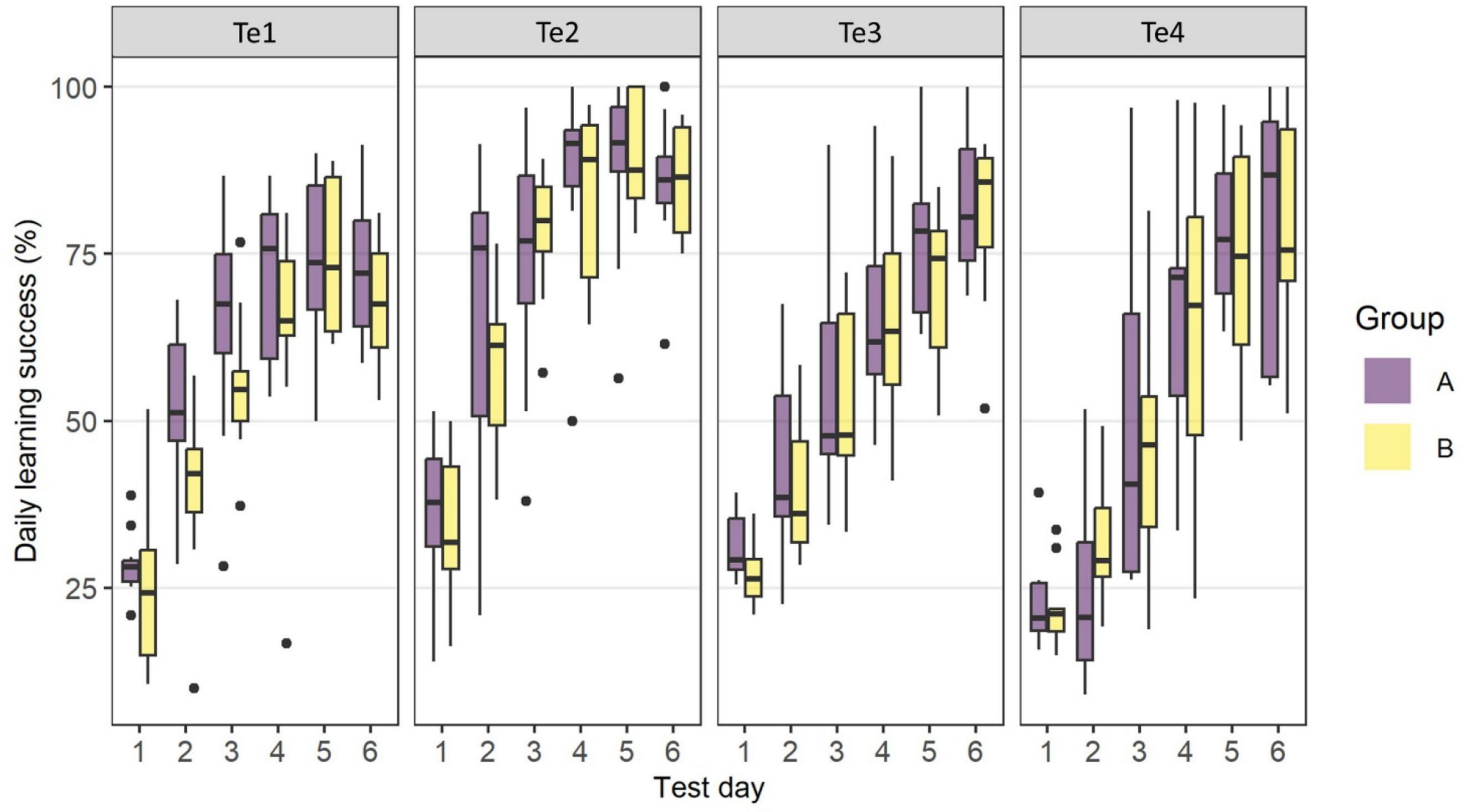
Learning curves for the six days of discrimination training in Te1–Te4 for Group A and Group B. The boxplots show the distribution of the data with the 25th, 50th (median), and 75th percentiles as coloured boxes, the 90th percentile as whiskers and black dots as outliers.

Figure 4 shows the learning curves for the 6 days of discrimination training. The two groups’ learning curves did not appear to differ significantly across tests. The animals reached the learning criterion of 46% correct choice on the 2nd (Te1 and Te2) and 3rd training days (Te3), respectively. Learning curves plateaued from day 4 for Te1 and 2, but increased steadily over the 6 days for Te3. In Te4, however, the learning curves of both groups increased only from Day 3 and the learning criterion was not reached before the 4th training day.

**Figure 5:**
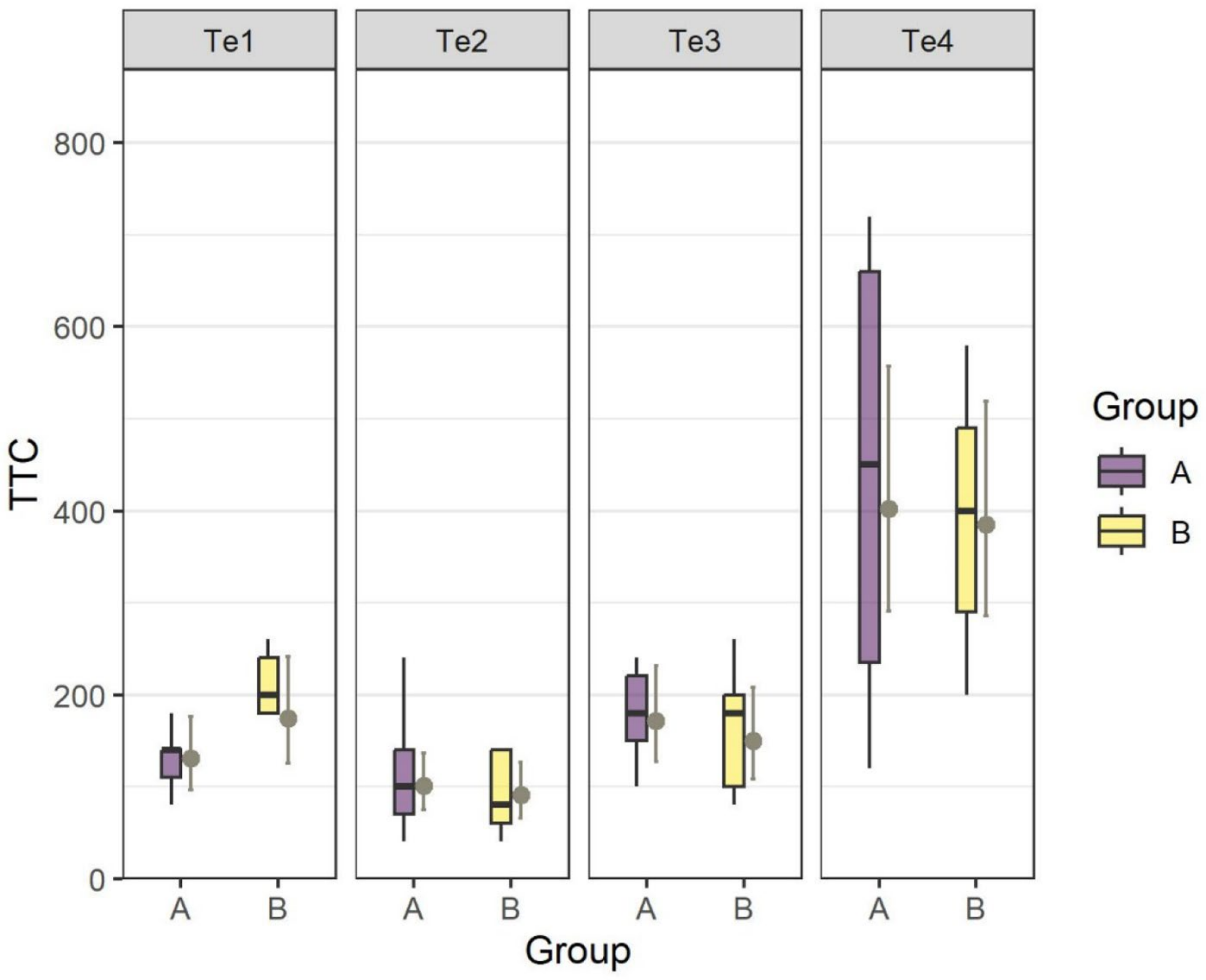
Estimated mean number of trials (TtC) to reach the learning criterion in Te1–Te4 for Group A and Group B. The boxplots show the distribution of the data with the 25th, 50th (median), and 75th percentiles as coloured boxes and the 90th percentile as whiskers. LSMs (±CL) are indicated in gray.

By running an ANOVA to test for the fixed-effects *Test* and *Group* of the fitted LMM, we found *Test* (Te1–Te4) had an impact on the absolute learning performance (*TtC*) (F_3,51.87_ = 39.72, *p* < 0.001) while there was no difference in *TtC* between groups in any of the four tests (Figure 5). The estimated number of *TtC* was 131 [Te1, CL (97, 176)], 101 [Te2, CL (75, 136), 171 [Te3, CL (127, 231), and 403 [Te4, CL (291, 557)] for Group A and 174 [Te1, CL (126, 241)], 91 [Te2, CL (66, 126)], 150 [Te3, CL (108, 208)] and 385 [Te4, CL (286, 519)] for Group B. *Post hoc* tests revealed no difference in *TtC* between Te1, Te2, or Te3, whereas the goats in both groups had to undergo more than twice as many trials to pass *TtC* in Te4 compared with the previous three tests.

## 4. Discussion

In the current study, we investigated whether goats are able to discriminate between familiar conspecifics from their own group and unfamiliar goats from an outgroup when presented with 2D photos on a computer screen, as has been shown previously for various bird and mammal species (Kendrick et al., 1995; Parr, 2003; Ferreira et al., 2004; Coulon et al., 2009; Leopold and Rhodes, 2010; Wilkinson et al., 2010; Talbot et al., 2016). We presented the same sets of portrait (Te1), profile (Te2), or headless body (Te3) photos of different goats as 4-choice discrimination tests to two experimental groups (A and B). We used photos of new goats in each test. In all tests, Group A animals had to discriminate a familiar goat from their own group from three unfamiliar goats from group B, while Group B animals had to discriminate one unfamiliar goat from group A from three familiar goats from group B. In Te1 to Te3, the animals in both groups learned the discriminations at the same rate within two to 3 days and needed between 91 and 174 trials to pass the TtC. Their learning performance was comparable to previous studies in which goats had to discriminate simple or complex symbols of the same size (Langbein et al., 2008). When goats were trained in successive discrimination tests of this type, we observed a continuous improvement in learning performance (Langbein et al., 2007). In the current study, learning performance increased in Te2 (decreased TtC) before decreasing slightly in Te3 (increased TtC). For the first two tests, we used portrait or profile photos, which provide visual cues of the face that has been proven to be particularly important for conspecific recognition (Parr et al., 2000; Sheehan and Tibbetts, 2011) and likely made it easier for goats to complete the tasks. In contrast, we used headless body photos in Te3 where these stimuli were missing. Finally, in the reversal test (Te4), in which the rule previously applied in Te1 to Te3 was reversed so that the rewarded photo now showed a goat from Group B and the three distractor pictures showed goats from Group A, TtC was more than twice as high as in the previous three tests, even though we again used portrait photos. We concluded that the animals were probably not guided by visual cues alone in learning the discriminations.

An important issue related to the social recognition of 2D representations of real individuals is whether animals learn to discriminate the photos merely as different visual stimuli without social meaning (Zayan and Vauclair, 1998), or whether they actually understand the photos as representations of real individuals (Pokorny and de Waal, 2009). According to Fagot et al. (2000, 2010). This would correspond to either the “low pathway” of processing 2D visual stimuli, in which the image are either processed as visual stimuli only, regardless of its content, or the image is confused with the real object. Alternatively, they may choose the “high pathway” of image processing, in which the image is “read” as a representation of the real object, suggesting that goats have the cognitive ability to process the images as reference stimuli (DeLoache, 2004).

A first indication that the goats in this study perceived the dual nature of the photos and processed them as referential symbols would have been a spontaneous preference for the photo of the familiar goat in Group A in PT1 to PT3, whereas Group B should have preferred an animal from its own group (one of the three distractor photos). Spontaneous preferences for 2D representations of familiar over unfamiliar subjects has been observed in nonhuman primates and other mammal and bird species (Parr and de Waal, 1999; da Costa et al., 2004; Coulon et al., 2010; Murai et al., 2011; Stephan et al., 2013; but see Dawkins, 1996; Ungerfeld et al., 2021). However, we did not find a clear preference for the photos of familiar conspecifics in PT1 to PT4 in neither of the two groups. In addition, the few preferences for specific photos in the different PTs were identical for both experimental groups. This could indicate that the goats in both groups learned to discriminate the photographs solely as visual stimuli without further reference to reality, using the same visual features in the images. The goats in this study were naïve about processing photos at the start of the experiment. Humans from cultures in which the photographic representation of objects or people is unknown do not spontaneously grasp the referential meaning of images (Deregowski, 2000) and children in Western societies begin to understand images as representations of reality only at the age of approximately 2.5 years, after appropriate experience with pictorial representations (DeLoache, 2004). An initial reaction of picture-naïve humans and animals to photos of real objects or subjects is often confusion. They attempt to touch or eat the pictured object, or they show signs of decreased fear or aggression when familiar or unfamiliar conspecifics are pictured (Ferreira et al., 2004; Parron et al., 2008; Vonk and Hamilton, 2014). Since we did not observe any such responses, we assume that the animals discriminated the photos at this stage of the experiment as visual stimuli independent of the real content of the images.

Another indication that animals understand photos as referential stimuli is that individuals that are asked to discriminate a photo of a familiar conspecific learn faster than animals that are asked to reliably select the photo of an unfamiliar conspecific (Kendrick et al., 1996; de Waal and Pokorny, 2008). In the current study, however, regardless of whether we used portrait, profile, or headless body photos, we were unable to demonstrate a difference in learning performance between the two test groups that would indicate that the rewarded photos were learned better by Group A than by Group B. Goats of both groups learned quickly to discriminate the small, complex and colourful photos. In this regard, it is worth noting that we used six slightly different shots of each goat in each test. High visual acuity in goats was demonstrated by Blakeman and Friend (1986) when they trained goats to discriminate between two 3.4 × 3.4 cm white letters, X and O, and found that they could tell the letters apart well at distances between 1.50 and 2.0 m. In previous studies, we have already shown that goats can learn a large number of symbols of varying complexity sequentially and then recall them simultaneously (Langbein et al., 2007, 2008). This indicates high visual learning flexibility (replacing former cues with new ones), memory capacity (a high number of cues that can be stored at any one time), and retention time (a high number of learned cues can be concurrently recalled after several weeks). However, the lack of differences in learning performance between the two groups in Te1 to Te3 again seems to indicate that the goats likely learned the discriminations based solely on visual features in the photos, but did not understand the referential nature of the stimuli. There is always a risk that continued exposure to pictures will reinforce the independence between image content and reality, with the consequence that the animal falls into the trap of discriminating photos based only on visual features rather than processing the referential image content (Fagot et al., 2010).

While transfer experiments are often used to demonstrate understanding of the referential meaning of 2D images of conspecifics based on the establishment of familiar and unfamiliar categories (Ferreira et al., 2004; Pokorny and de Waal, 2009; Coulon et al., 2010), we conducted a reversal learning experiment. In Te4, we again used portrait photos in which most of the features important for IR could be found (Behrmann and Avidan, 2022). If the goats had discriminated the photos as visual stimuli without any reference to reality, we would have expected a comparable or even better learning performance as in Te1 to Te3, regardless of the fact that we reversed the rewarded category for both groups (Langbein et al., 2007). In contrast, the goats of both groups needed more than twice as many trials as in the first three tests to learn the discrimination. This is clear evidence that over the course of Te1 to Te3, the goats in both groups had established familiar and unfamiliar categories and finally understood the referential relationship between the 2D photos of conspecifics and real individuals. The design of the discrimination tasks in Te1 to Te3 repeatedly required Group A goats to learn to select the photo of a goat from a familiar conspecific in their own group, while Group B goats repeatedly selected the photo of an unfamiliar goat. After reversing this rule in Te4, the goats in both groups apparently had to overcome this previously learned rule before they could begin learning the reversed contiguity. This can be seen from the learning curves in Te4, where the goats of both groups could only slightly increase learning success on the first two test days and only reached the learning criterion on the 4th day. Regarding the absolute learning performance, the goats needed more than twice as many trials in Te4 compared to Te1 to Te3 to reach the learning criterion. The most likely explanation for this decline in learning performance is that the animals established the categories familiar and unfamiliar and the associated rule of which photo to choose as they progressed through Te1 to Te3, and applying this rule in Te4 first failed. One aspect that was likely to have contributed significantly to the establishment of a reference relationship between the photographs and the real conspecifics was probably the use of six slightly different photographs of the individual goats in the various tests. While it was not easy for the goats to orient themselves to recurring visual stimuli in the individual photos, this slight variation in the photos possibly led to the goats establishing reference to the real animals.

We can only speculate on why we did not find spontaneous preferences for the photos of known conspecifics in the preference tests or higher learning performance when discriminating a photo of a familiar goat compared to an unfamiliar one as previously shown (da Costa et al., 2004; Coulon et al., 2010). A possible reason for this could be the variation in the representation of the animals in the photos in the different tests (portrait, profile or headless body photos). The goats had to repeatedly adapt to new visual stimulus combinations in the different discrimination tests, which required repeated relearning in the first three tests and in the associated preference tests rather than showing *ad-hoc* preferences or quickly using established learning content. However, this variation in the representation of conspecifics in the photos in the different tests, together with the variation in the photos of individual animals in each test, was probably the key to understanding the referential relationship between the photo and real animals in the goats.

## Data availability statement

The raw data supporting the conclusions of this article will be made available by the authors, without undue reservation.

## Ethics statement

The animal study was reviewed and approved by Committee for Animal Use and Care of the Ministry of Agriculture, Environment and Consumer Protection of the federal state of Mecklenburg-Vorpommern, Germany (Ref. 7221.3-2-014/20).

## Author contributions

JL conceptualized the study and wrote the main part of the manuscript. KS and JL collected the data. MM-Z analysed the data. All authors contributed to the article and approved the submitted version.

## Conflict of interest

The authors declare that the research was conducted in the absence of any commercial or financial relationships that could be construed as a potential conflict of interest.

## Publisher’s note

All claims expressed in this article are solely those of the authors and do not necessarily represent those of their affiliated organizations, or those of the publisher, the editors and the reviewers. Any product that may be evaluated in this article, or claim that may be made by its manufacturer, is not guaranteed or endorsed by the publisher.
